# Editorial: Advances in Craniofacial and Dental Materials Through Nanotechnology and Tissue Engineering

**DOI:** 10.3389/fphys.2019.00303

**Published:** 2019-03-27

**Authors:** Giovanna Orsini, Angelo Putignano, Thimios A. Mitsiadis

**Affiliations:** ^1^Orofacial Development and Regeneration, Centre for Dental Medicine, Institute of Oral Biology, University of Zurich, Zurich, Switzerland; ^2^Department of Clinical Sciences and Stomatology, Polytechnic University of Marche, Ancona, Italy

**Keywords:** tooth, craniofacial, regeneration, stem cells, tissue engineering, nanotechnology, bone, dental

The extraordinary recent advances in biomaterials, tissue engineering, and nanotechnology sciences opened new horizons and now offer innovative options to the clinical practitioners for the restoration and/or regeneration of missing and damaged dental and bone tissues (Mitsiadis et al., 2015; Orsini et al., 2018). Dental and craniofacial treatments are commonly based on specific biomaterials. Substantial improvements of such biomaterials have been partly achieved via incorporation of filler elements possessing at least one dimension in the nanometer range for the enhancement of the healing capability of the tissues (Yi et al., 2016). Nanometric surface modifications of biomaterials are constantly applied in order to ameliorate their physical and biological properties, thereby improving the quality and the duration of tissue repair (Variola et al., 2011). The structure and functionality of dental and periodontal tissues are compromised upon traumatic and carious lesions, periodontal diseases and genetic disorders. Therefore, there is a high medical need for the regeneration and/or repair of damaged teeth and the surrounding alveolar bone. Although bone and several of the dental-specific mineralized tissues such as dentin and cementum exhibit a natural regenerative potential, tooth enamel cannot be regenerated in humans (Mitsiadis et al., 2015). In fact, the enamel-producing ameloblastic cells are eliminated soon after the eruption of teeth in the oral cavity and therefore the regeneration of enamel is impossible upon its damage by the various tooth insults. However, in several teeth of other species, such as in incisors of rodents, enamel is continuously regenerated. Studies in rodent incisors allow the collection of precious information fundamental to elucidate the molecular and cellular mechanisms that are involved in the regeneration of enamel. This information might be useful for tooth enamel repair in humans, when combined with the recent technological advances in nanosciences, tissue engineering, and stem cell biology, and it will undoubtedly bring in the near future extremely innovative dental treatments in the clinics.

The present research topic focuses on the use of different modern materials coupled with nanotechnology to improve the clinical performances in all fields of dentistry as well as in bone regeneration procedures. Prominent researchers within the craniofacial and dental fields have contributed with important discoveries and generated exciting results concerning the repair or regeneration of the mineralized tissues using biomaterials.

Tooth specific proteins such as Amelogenin and Enamelin are involved in the initial steps of enamel formation (amelogenesis) that is followed by enamel mineralization, which is a very complex process. The role of Leucine Rich Amelogenin Peptide (LRAP), a product of the amelogenin gene, in enamel mineralization is examined (Le Norcy et al.) As full-length amelogenin, LRAP has been shown to regulate hydroxyapatite (HAP) crystal formation depending on its phosphorylation status. Micro-computed tomography, field emission scanning electron microscopy, transmission electron microscopy, diffraction imaging, and qPCR analysis have been used to elucidate the impact of its phosphorylation status on enamel mineralization. The results showed that LRAP, independently of its phosphorylation status, is able to induce an up-regulation of *Amelogenin* transcription and promote mineral formation in both cells and mouse molar germ cultures. Therefore, LRAP isoforms can be envisioned as potential candidates for the treatment of enamel lesions or defects.

The functions and roles of the extracellular matrix (ECM) molecules in the mineralization process of the various craniofacial tissues are also under intensive investigation. The ECM protein TRIP-1 may play a pivotal role in the organization of the mineralized matrices of bone and dentin (Chen and George). TRIP-1 regulates both osteogenesis and angiogenesis, which are essential biological processes for the elaboration of novel therapeutic approaches focusing on mineralization-related disorders.

Although bone has an amazing ability to heal spontaneously, in several specific cases it fails to heal. Hence, it is important to consider the mechanical conditions affecting its regeneration and the influence of the environment. Since craniofacial morphology is regulated genetically and epigenetically by microenvironmental factors, the effects of food consistency in the mandibular morphology of mice have been assessed (Kono et al.) Micro-3D CT data has been used to detect the 3D quantitative changes occurring in the mandibular skeletal structures and the site-specific bone response to different masticatory activities.

The application of mechano-biological principles such as the significance of fixation and controlled motion (dynamization) for enhancing bone healing is of prime importance (Glatt et al.) The selection of appropriate fixation devices, scaffolds, signaling molecules is mandatory for achieving consistent and predictable bone healing that guarantees the clinical outcome. For instance, the tension that is created by gradual mechanical distraction stimulates the formation of new bone, vessels, nerves, and muscles, thus leading to bone lengthening and regeneration. Although recent research has unraveled the interplay between mechanics and biology, the realization of *in vivo* studies is compromised due to the lack of important tools. Deficiencies in controlling the chemical, physical, and biological environment within the healing defects are still of actuality.

Scaffolds are key elements for the success of tissue regeneration. Dental implantology has benefited from recent advances in bone regeneration, due to the use of many osseous substitutes that can be clinically applied in regenerative approaches, such as sinus augmenting procedures. A biomimetic porous three-dimensional MgHA/collagen-based scaffold could be used for enhancing the poor quantity and/or quality of bone in delicate maxillary areas (e.g., nearby the sinus) where the placement of dental implants is envisaged (Scarano et al.) Tomographic, radiological, histological and histomorphometric analyses have demonstrated the enhancement of bone formation and the complete resorbing of the scaffold after 6 months of treatment.

Various diseases affecting teeth or their supporting tissues lead to their infection and progressive destruction and loss. Guided tissue regeneration is a procedure widely used for the treatment of severe periodontal diseases. This technique involves the placement of an occlusive barrier to facilitate the regeneration of damaged areas by periodontal ligament stem cells (PDLSCs). An ECM-based scaffold has been compared to a Collagen I (COLI) membrane for its ability in providing a suitable microenvironment that will support proliferation and proper differentiation of PDLSCs (Wang et al.) The cementoblastic/osteogenic activity of PDLSCs was characterized by evaluating periodontal tissue differentiation biomarkers, including TNAP enzyme activity, Alizarin red stain, and differential mRNA expression. The ECM membrane exhibited superior biological properties when compared to COLI membrane, and significantly promoted PDLSCs cell proliferation, differentiation and mineral formation. *In vivo* studies are still required to confirm these findings and finally offer new alternatives for the treatment of periodontal diseases using ECM membranes.

A new biomaterial for regenerative purposes has been proposed for endodontic treatment purposes (Hertig et al.) Scanning electron microscopy and spectrophotometry has been used to assess a fibrin hydrogel, a common scaffold employed for filling the pulp chamber, via an iodine-based radiopaque agent. The study has been performed in chick chorioallantoic membranes (CAM) of fertilized eggs and showed that the tested biomaterial is not toxic and allows monitoring of its degradation-related modifications.

The multidisciplinary strategies based on the association of stem cells with advanced tissue engineering products try to tackle a good number of challenges related to regenerative dentistry (Orsini et al.) Novel platforms for tooth modeling and inspired discoveries for diagnostic and therapeutic purposes have been advancing. The employment of nanostructured biomaterials is also decisive in the fine modulation of stem cell behavior in order to drive proper dental and bone tissue regeneration by providing proper tissue innervation and vascularization (Abdal Dayem et al., 2018). Similarly, nanotechnology-related achievements had a tremendous impact on the evolution of biomaterials, thus widening the possibilities for novel and more accurate treatments for the repair or replacement of damaged or lost enamel and dentin. The precise and efficient repair of these tissues has been greatly improved thanks to the introduction of novel and sophisticated composite and ceramic materials, as well as modern adhesive systems that may durably bond these materials to the dental tissues, thus increasing the longevity of tooth crown restorations (Monterubbianesi et al.) Nano-based synthetic resin composite materials, characterized by the employment of nanocluster fillers that enhance their physical properties, have been introduced over the last decade. This allowed the elaboration of simplified procedures for filling dental cavities in molars. The “bulk -fill composites” have been demonstrated to maintain a high degree of conversion and exhibited an incredible hardness both at the surface and base of any restoration, which is advantageous for numerous clinical cases.

Advanced imaging techniques can verify and quantify the degree of mineralization and vascularization of the implanted scaffolds at the earliest stages of bone regeneration (Giuliani et al.) Synchrotron produces brilliant X-ray photon beams, thus achieving a higher image quality with sub-micrometer spatial resolution. X-ray imaging and holotomography are suitable for detecting both *in vitro* and *ex vivo* the newly formed extracellular matrices, the early phases of hard tissue mineralization and the vascular network in regenerating bones.

Nanotechnological advances have also been largely implemented in diagnostic research, and these innovations could be also used in the field of oral oncology (Mascitti et al.) Non-invasive diagnostic devices constitute great promises for this field and the early treatment of oral cancers. Light-based detection systems have been introduced for earlier detection of oral squamous cell carcinomas and other potentially malignant disorders. Implementing light-based detection systems based on tissue-marking dyes and nanoparticle properties could be exploited for diagnosis and possible cure of several cancers.

The recent progresses in the fields of nanotechnology, tissue engineering, and stem cell biology allowed the generation of novel sophisticated biomaterials that could be successfully used for medical purposes. However, several concerns still exist concerning their applicability in the dental clinics. Therefore, there is an urgent need for clinical trials that will demonstrate the reliability and accuracy of these alternative strategies for tissue regeneration. Nevertheless, all these novel biomaterials and technological tools, combined with the constantly increasing scientific knowledge, offer exciting perspectives for modern alternative therapies in the dental and craniofacial fields.

## Author Contributions

All authors listed have made a substantial, direct and intellectual contribution to the work, and approved it for publication.

### Conflict of Interest Statement

The authors declare that the research was conducted in the absence of any commercial or financial relationships that could be construed as a potential conflict of interest.
